# Intraspecific variation in the turtle barnacle, *Cylindrolepas sinica* Ren, 1980 (Cirripedia, Thoracica, Coronuloidea), with brief notes on habitat selectivity

**DOI:** 10.3897/zookeys.327.5732

**Published:** 2013-08-30

**Authors:** Ryota Hayashi

**Affiliations:** 1 International Coastal Research Center, Atmosphere and Ocean Research Institute, The University of Tokyo, 5-1-5, Kashiwanoha, Kashiwa, Chiba, 277-8564, Japan; 2 Current address: Seikai National Fisheries Research Institute, Fisheries Research Agency 1551–8, Tairamachi, Nagasaki, 851–2213, Japan

**Keywords:** Turtle barnacle, *Cylindrolepas*, epibiont, variation, redescription, mitochondrial genes 12S and 16S

## Abstract

Specimens of the turtle barnacle *Cylindrolepas sinica* Ren, 1980 were collected from sea turtles in Japanese waters. The specimens were hexagonal in shape and were found burrowing into the sea turtle plastron. Specimens were dissected and the hard and soft parts were compared with the original description.

## Introduction

Ren (1980) described a new species of barnacle, *Cylindrolepas sinica*, collected from green sea turtles, *Chelonia mydas* (Linnaeus, 1758). Hayashi (2009) subsequently recorded *Cylindrolepas sinica* from three species of sea turtle: the green sea turtle, *Chelonia mydas*, loggerhead, *Caretta caretta* (Linnaeus, 1758), and hawksbill, *Eretmochelys imbricata* (Linnaeus, 1766). Subsequently, 13 species of the superfamily Coronuloidea, including *Cylindrolepas sinica*, were recorded during a 10-year (2002–2011) survey of epibionts attached to marine vertebrates from Japanese waters (Hayashi 2012). As shown in previous studies (Hayashi 2009, 2012), *Cylindrolepas sinica* is a common species on Japanese sea turtles.

**Figure 1.**
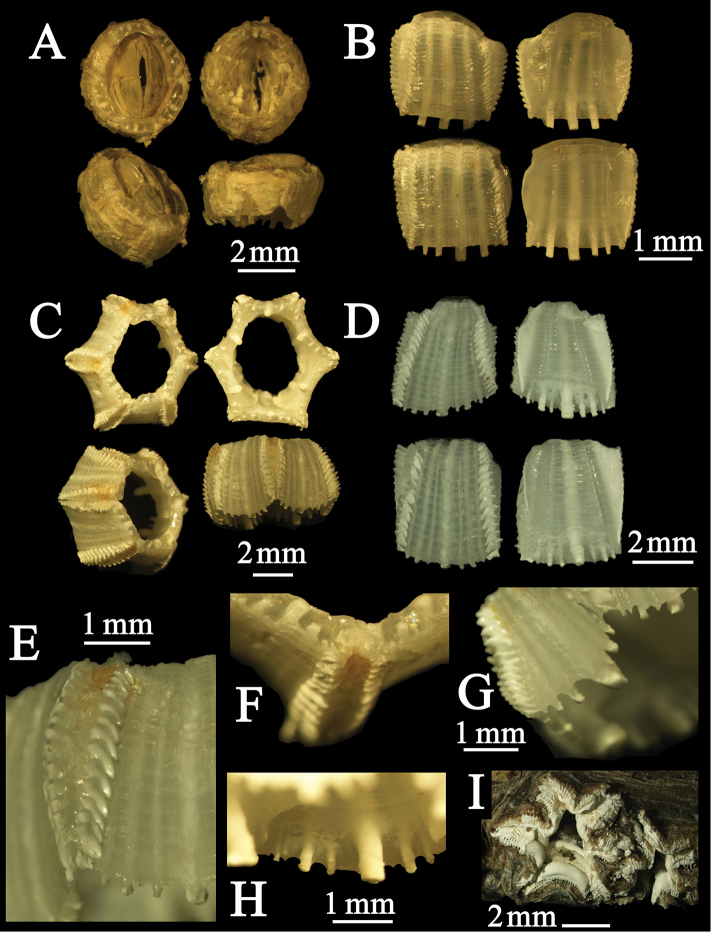
*Cylindrolepas sinica* Ren, 1980. **A–B** original variant occurring on the soft skin of sea turtles (RUMF-ZC-02030) **C–H** hexagonal variant occurring on the plastron of sea turtles (RUMF-ZC-02045) **A** and **C**, upper, basal, upper oblique and lateral views **B** and **D** parietal plates (surface and back view) **E** sutural elaboration of the parietal wall **F** upper view of radii **G** inner view of basal margin **H** basal margin with parietal ribs **I** hexagonal shell wall of *Xenobalanus globicipitis*.

This species was described as a cylindrical and rounded barnacle in previous studies (as shown in Fig. 1A and 1B). This study describes the intraspecific variation occurring in *Cylindrolepas sinica* and emphasises the morphological differences between *Cylindrolepas sinica* and related species. Brief comments on host selectivity are also presented.

## Materials and methods

Epibiotic barnacles were sampled from sea turtles on breeding beaches, in bycatch, and from strandings in Japanese waters (see Hayashi 2012). Specimens of *Cylindrolepas sinica* were collected from the turtle (skin and plastron) and preserved in 99% ethanol. The specimens were dissected and their soft parts mounted on slides with a drop of glycerine. The specimens examined have been deposited in Fujukan, the Museum of the University of Ryukyus, under accession numbers RUMF-ZC.

## Systematics

### Class Maxillopoda Dahl, 1956
Subclass Cirripedia Burmeister, 1834
Superorder Thoracica Darwin, 1854
Order Sessilia Lamarck, 1818
Suborder Balanomorpha Pilsbry, 1916
Superfamily Coronuloidea Newman & Ross, 1976
Family Platylepadidae Newman & Ross, 1976
Genus *Cylindrolepas* Pilsbry 1916

#### Cylindrolepas sinica

Ren, 1980

http://species-id.net/wiki/Cylindrolepas_sinica

Figs 1 , 2

Cylindrolepas sinica Ren, 1980: 194, fig. 6; pl. 2 figs 12–20. Hayashi 2009: 1, fig. 1A, B. Hayashi 2012: 118, figs 10, 15g, pl. 3d.
Platylepas decorata Zardus & Balazs, 2007: 1303, figs 7–9. Frick and Zardus 2010: 294.

##### Material examined.

From the plastron of a green sea turtle stranded on Ishigaki Island, Okinawa, Japan, November 25, 2001, Ryota Hayashi coll. (RUMF-ZC-02047); from the plastron of a living green sea turtle, April 17, 2004, Hahajima Island, Ogasawara, Tokyo, Japan, Ryota Hayashi coll. (RUMF-ZC-02045); from a plastic tag on a stranded loggerhead sea turtle, September 26, 2010 at Yomitan, Okinawa, Japan, Ryota Hayashi coll. (RUMF-ZC-02048).

##### Additional description of intraspecific variation in the parietal wall.

Wall outline stellate, parietes concave (Fig. 1C); translucent between external ornamentation, external longitudinal ridges low, broad, poorly defined, growth ridges numerous, fine, closely spaced; (Fig. 1D); sutural elaborations opaque, erect, irregular ridges slightly directed toward apex, not cupped (Fig. 1E); radii very narrow, externally teeth partly concealed by sutural elaborations (Fig. 1F); internal midrib broad, short, flaring terminally or club-shaped, directed more downward than medially (Fig. 1G); internal lateral ribs well developed, moderately broad, short, extending below the basal margin, approximately same size and number of ribs on each side of midrib on all plates (Fig. 1G–H); sheath about two-thirds height of wall, basally terminating abruptly, not depending (Fig. 1D). Opercular valves and soft parts as described in Ren (1980) and Hayashi (2012).

##### Remarks.

**Figure 2.**
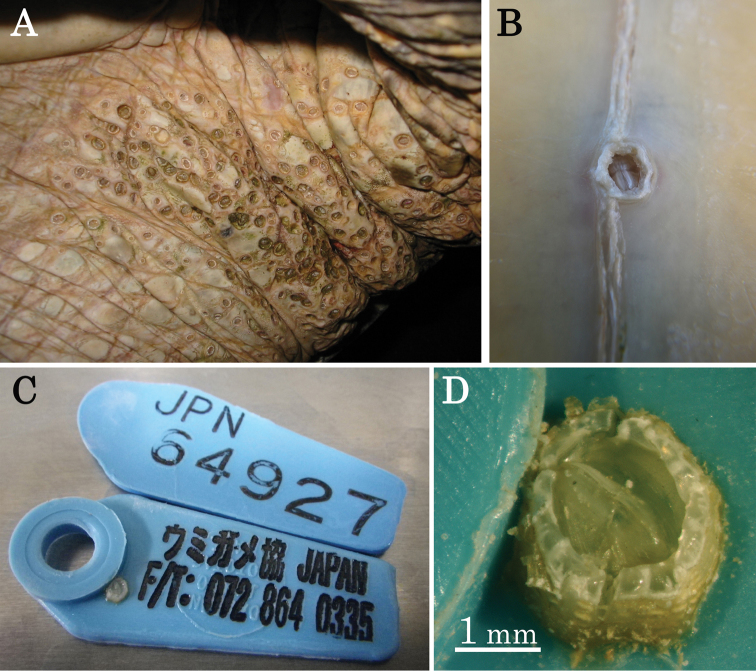
Habitat of *Cylindrolepas sinica* Ren, 1980. **A** the original rounded form aggregated on the tail of a green sea turtle **B** the hexagonal variant burrowing into a green sea turtle plastron **C** an unusual specimen attached to a plastic tag on a loggerhead sea turtle **D** close-up view of the individual attached to the plastic tag.

The original description of *Cylindrolepas sinica* described the rounded, cylindrical form and is accurate for individuals occurring on the soft skin of sea turtles. The general morphology of this species is as described by Ren (1980) and Hayashi (2012) and illustrated in the present work in Figs 1A, 1B, and 2A. Frick and Zardus (2010) and Frick (2013) regarded *Cylindrolepas sinica* as a junior synonym of *Platylepas decorata* Darwin, 1854. However, morphological differences between *Cylindrolepas sinica* and *Platylepas decorata* are clearly detailed by Monroe and Limpus (1979), Ren (1980), Young (1991), and Hayashi (2012). *Cylindrolepas sinica* can be distinguished fromother species easily by the morphological characteristics listed in Table 1. In addition, the mitochondrial sequence variation of this and related species has been confirmed (the 12S rRNA, tRNA-Val and 16S rRNA regions, Table 2, see Appendix). Therefore, *Cylindrolepas sinica* is a valid species. In the phylogenetic analysis of Hayashi et al. (2013), *Cylindrolepas sinica* clustered with the whale barnacles (*Xenobalanus*, *Coronula*, and *Cryptolepas*). The pseudo-stalked barnacle *Xenobalanus globicipitis* also has hexagonal and cylindrical shell walls (Fig. 1A–I). Comparing these findings, *Cylindrolepas sinica* is likely ancestral to the whale barnacles (Hayashiet al. 2013).

**Table 1.** Comparative features of *Cylindrolepas* spp. and *Platylepas* spp.

	**Labrum**	**Basal margin of sheath**	**Ornamentation of suture**	**Longitudinal ridges on parietes**	**Radii**	**Midrib folds**	**Secondary ribs**	**Inner surface of parietes**
*Cylindrolepas sinica*	with a few teeth on each crest	continuous with inner laminae	present	absent	not visible	not visible	present	smooth
*Cylindrolepas darwiniana*	multidentate	continuous with inner laminae	rudimentary	rudimentary	not visible	not visible	present	smooth
*Platylepas decorata*	multidentate	depending	present	present	not visible	conspicuous	present	smooth
*Platylepas hexastylos*	with a few teeth on each crest	depending	absent	absent	visible, narrow	conspicuous	absent	with longitudinal ridges

**Table 2.** List of the materials examined. The GenBank accession numbers are from Hayashi et al. (2013).

	**Host animal**	**Collected Locality**	**Materials deposited number**	**GenBank accession numbers**
*Cylindrolepas sinica* (hexagonal form)	*Chelonia mydas*	Hahajima I., Ogasawara, Tokyo	RUMF-ZC-02045	AB723955
*Cylindrolepas sinica* (hexagonal form)	*Chelonia mydas*	Hahajima I., Ogasawara, Tokyo	RUMF-ZC-02047	AB723954
*Cylindrolepas sinica* (rounded form)	*Chelonia mydas*	Kanna, Ginoza, Okinawa	RUMF-ZC-02030	AB723953
*Cylindrolepas darwiniana*	*Caretta caretta*	Toya, Yomitan, Okinawa	RUMF-ZC-02029	AB723959
*Cylindrolepas darwiniana*	*Caretta caretta*	Toya, Yomitan, Okinawa	RUMF-ZC-02028	AB723960
*Platylepas decorata*	*Chelonia mydas*	Kanna, Ginoza, Okinawa	RUMF-ZC-02042	AB723950
*Platylepas decorata*	*Chelonia mydas*	Kanna, Ginoza, Okinawa	RUMF-ZC-02046	AB723951
*Platylepas decorata*	*Chelonia mydas*	Kanna, Ginoza, Okinawa	RUMF-ZC-02027	AB723952
*Platylepas hexastylos*	*Caretta caretta*	Otsuchi, Iwate	RUMF-ZC-02025	AB723956

## Discussion

The rounded form (Fig. 1A and 1B) of this species (described by Ren 1980; Hayashi 2012) is found in the soft skin of sea turtles and forms colonies in proximity to other individuals, as well as in aggregations (Fig. 2A). The hexagonal variant of this species burrows into the hard parts of the turtle body (plastron) and is often found as isolated individuals (Fig. 2B). Therefore, the shell morphology of *Cylindrolepas sinica* exhibits phenotypic plasticity through habitat selection.

In a rare case, one individual was collected from a plastic tag attached to a loggerhead sea turtle (Fig. 2C, D). The tagged turtle was captured on June 24, 2010 in a set net at Yomitan, Okinawa, Japan, and recaptured in the same net on August 26, and finally found as a floating stranding nearby on September 26. These records indicate that this turtle was a resident in this coastal area, as reported in Hayashi (2009), and the presence of *Cylindrolepas sinica* is consistent with the previous report. In addition, this is the first record of the occurrence of *Cylindrolepas sinica* on an artificial object. This case indicates that *Cylindrolepas sinica* can attach to hard substrates, as well as living soft tissue, and the host selectivity of *Cylindrolepas sinica* is not the substrate material. Nogata and Matsumura (2006) reported the larval development and settlement of the whale barnacle, *Coronula diadema* (Linnaeus, 1767), which settled in a polystyrene Petri dish containing a small, isolated piece of skin tissue from the host whale. They suggested the involvement of a chemical cue from the host whale tissue in inducing larval settlement. In light of these findings, our finding of *Cylindrolepas sinica* settling on a plastic tag, and not directly on the body of the turtle, suggests that this settlement was triggered by a similar chemical cue. More information is necessary to clarify the settlement mechanism of epibiotic barnacles.

## Supplementary Material

XML Treatment for Cylindrolepas sinica

## Figures and Tables

**Figure:**
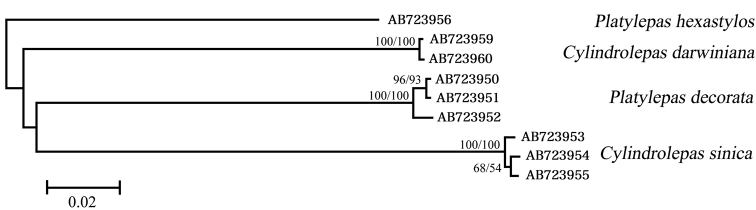
The consensus trees in the neighbour-joining and maximum-parsimony analyses were inferred from 1000 bootstrap replicates and both analyses were conducted using MEGA ver. 5.0 (Tamura et al. 2011). The sequences were aligned using MUSCLE included in MEGA ver. 5.0. At each node, the numbers separated by the slash indicate the percentage NJ and MP bootstrap support, respectively (1000 replicates).

## Appendix 1

Tables

## Appendix 2

Supplementary file

## References

[B1] Frick MG (2013) A rejoinder and addendum to Hayashi (2011) regarding the systematics and biology of the turtle and whale barnacles (Cirripedia: Balanomorpha: Coronuloidea). Journal of the Marine Biological Association of the United Kingdom 93(1): 183-187. doi: 10.1017/S0025315412000471

[B2] Frick MG Zardus JD (2010) First authentic report of the turtle barnacle *Cylindrolepas darwiniana* since its description in 1916. Journal of Crustacean Biology 30: 292-295. doi: 10.1651/09-3161.1

[B3] Hayashi R (2009) New host records of the turtle barnacle, *Cylindrolepas sinica*: a case study of sea turtles’ behaviour and their epibionts. Marine Biodiversity Records 2: e165. doi: 10.1017/S1755267209990947

[B4] Hayashi R (2012) Atlas of the barnacles on marine vertebrates in Japanese waters including taxonomical review of superfamily Coronuloidea (Cirripedia: Thoracica). Journal of the Marine Biological Association of the United Kingdom 92(1): 107-127.

[B5] Hayashi R Chan BKK Simon-Blecher N Watanabe H Guy-Haim T Yonezawa T Levy Y Shuto T Achituv Y (2013) Phylogenetic position and evolutionary history of the turtle and whale barnacles (Cirripedia: Balanomorpha: Coronuloidea). Molecular Phylogenetics and Evolution 67(1): 9-14. doi: 10.1016/j.ympev.2012.12.018 23306306

[B6] Linnæus C (1758) Systema naturæ per regna tria naturæ, secundum classes, ordines, genera, species, cum characteribus, differentiis, synonymis, locis. Tomus I Editio decima, reformata, [1-4], 1–824. Holmiæ. (Salvius).

[B7] Linnæus C (1766) Systema naturæ per regna tria naturæ, secundum classes, ordines, genera, species, cum characteribus, differentiis, synonymis, locis. Tomus I Editio duodecima, reformata, 1–532. Holmiæ. (Salvius).

[B8] Monroe R Limpus CJ (1979) Barnacles on turtles in Queensland waters with descriptions of three new species. Memoirs of the Queensland Museum 19: 197-223.

[B9] Nogata Y Matsumura K (2006) Larval development and settlement of a whale barnacle. Biology Letters 2: 92-93. doi: 10.1098/rsbl.2005.0409 17148335 PMC1617185

[B10] Ren X (1980) Turtle barnacles of the Xisha Islands, Guangdong Province, China. Studia Marina Sinica 17: 187-197.

[B11] Tamura K Peterson D Peterson N Stecher G Nei M Kumar S (2011) MEGA5: Molecular Evolutionary Genetics Analysis Using Maximum Likelihood, Evolutionary Distance, and Maximum Parsimony Methods. Molecular Biology and Evolution 28: 2731-2739. doi: 10.1093/molbev/msr121 21546353 PMC3203626

[B12] Young PS (1991) The superfamily Coronuloidea Leach (Cirripedia, Balanomorpha) from the Brazilian coast, with redescription of *Stomatolepas* species. Crustaceana 61: 190-212. doi: 10.1163/156854091X00678

[B13] Zardus JD Balazs GH (2007) Two previously unreported barnacles commensal with the green sea turtle, *Chelonia mydas* (Linnaeus, 1758), in Hawaii and a comparison of their attachment modes. Crustaceana 80: 1303-1315. doi: 10.1163/156854007782605547

