# Supplementary material for: Intraspecific variation in the turtle barnacle, Cylindrolepas sinica Ren, 1980 (Cirripedia, Thoracica, Coronuloidea), with brief notes on habitat selectivity
Source: Zookeys. 2013 Aug 30;(327):35–42. doi: 10.3897/zookeys.327.5732 (PMC3807745; doi:10.3897/zookeys.327.5732)
Supplement: Supplementary file 3 — Supplementary file [file ZooKeys-327-035-s002.pdf]

Supplementary file 1

Phylogenetic analysis

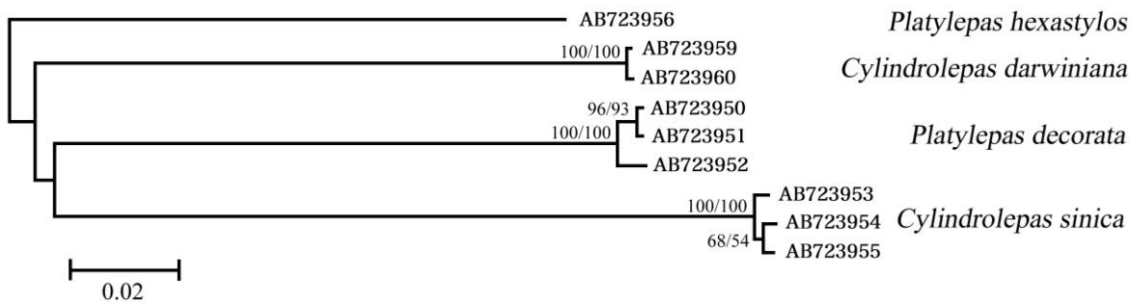

The consensus trees in the Neighbor Joining and Maximum Parsimony analyses were inferred from 1000 bootstrap replicates and both analyses were conducted in MEGA v5.0 (Tamura et al., 2011). The sequences were aligned using MUSCLE included MEGA v5.0. At each node, the numbers with the slash indicate the percentage of NJ and MP bootstrap support (1000 replicates).

Tamura K, Peterson D, Peterson N, Stecher G, Nei M, Kumar S (2011) MEGA5: Molecular Evolutionary Genetics Analysis Using Maximum Likelihood, Evolutionary Distance, and Maximum Parsimony Methods. Molecular Biology and Evolution, 28: 2731-2739.
